# Glucagon-like Peptide-1 Receptor Agonists Are Not Associated with Increased Risk of Acute Pancreatitis or Pancreatic Complications, Including Pancreatic Cancer, in Type 2 Diabetes: A Global Propensity-Matched Cohort Study

**DOI:** 10.3390/jpm16090478

**Published:** 2026-09-16

**Authors:** Yan Sun, Benjamin Liu, Hanyou Loh, Samantha Mathialagan, Cindy Hsin-Ti Lin, Yasir Tarabichi, Donovan Veccia, Richard Wong, William Tse, Gengqing Song

**Affiliations:** 1 Department of Medicine, MetroHealth Medical Center and Case Western Reserve University, Cleveland, OH 44106, USA; ysun1@metrohealth.org (Y.S.); benjamin.liu1@nm.org (B.L.); smathialagan@metrohealth.org (S.M.); linh4@ccf.org (C.H.-T.L.); 2 Department of Internal Medicine, Canton Medical Education Foundation, Canton, OH 44710, USA; hanyou.loh@aultman.com; 3 Center for Clinical Informatics Research and Education, MetroHealth Medical Center and Case Western Reserve University, Cleveland, OH 44106, USA; ytarabichi@metrohealth.org; 4 Division of Gastroenterology and Hepatology, MetroHealth Medical Center and Case Western Reserve University, Cleveland, OH 44106, USA; dveccia@metrohealth.org (D.V.); rwong1@metrohealth.org (R.W.); 5 Gene and Cell Therapy Institute, MetroHealth Medical Center and Case Western Reserve University, Cleveland, OH 44106, USA; wtse@metrohealth.org; 6 Division of Medical Oncology, MetroHealth Medical Center and Case Western Reserve University, Cleveland, OH 44106, USA

**Keywords:** GLP-1 receptor agonists, type 2 diabetes mellitus, acute pancreatitis, biliary pancreatitis, chronic pancreatitis, pancreatic cancer

## Abstract

**Introduction:** Association between glucagon-like peptide-1 receptor agonists (GLP-1 RAs) and pancreatitis and its long-term complications remains controversial. Using a global electronic health record database, we evaluated pancreatic outcomes following GLP-1 RA initiation in adults with type 2 diabetes mellitus (T2DM). **Methods:** Adults aged ≥18 years who were alive at the index date; T2DM patients initiating GLP-1 RAs between 2013 and 2025 were identified in the TriNetX network and compared with patients initiating sodium–glucose cotransporter-2 inhibitors (SGLT2i). Sensitivity analyses included comparisons with other second-line diabetes therapies, excluding dipeptidyl peptidase-4 inhibitors, and analyses were restricted to U.S.-based health care organizations. Patients with pre-existing pancreatic conditions were excluded. Cohorts were propensity score-matched for treatment indications, pancreatitis risk factors, and demographics. Kaplan–Meier and Cox proportional hazard models evaluated acute pancreatitis (overall and biliary), chronic pancreatitis, pancreatic pseudocyst, and pancreatic cancer. **Results:** After matching, 291,152 patients were included in each cohort. GLP-1 RA initiation was not associated with an increased hazard of overall acute pancreatitis compared with SGLT2i initiation (HR 1.095; 95% CI 0.996–1.204), with concordant Kaplan–Meier analyses. A modest, etiology-specific increase in biliary acute pancreatitis was observed in the primary comparison (HR 1.276; 95% CI 1.010–1.612) but was not consistent across sensitivity analyses. No differences were observed in the risks of pancreatic pseudocyst (HR 0.729; 95% CI 0.525–1.014) or pancreatic cancer (HR 1.069; 95% CI 0.940–1.215). Results were similarly consistent in sensitivity analyses. **Conclusion:** GLP-1 RAs are not associated with an increased risk of acute pancreatitis, pancreatitis-related complications, or pancreatic cancer in T2DM adults; a modest biliary pancreatitis signal was observed, but was not consistent across sensitivity analyses.

## 1. Introduction

Glucagon-like peptide-1 (GLP-1) receptor agonists have become a cornerstone of therapy for type 2 diabetes mellitus (T2DM) and obesity, providing effective glycemic control, weight reduction, and cardiovascular and renal risk benefits [1]. With their rapidly expanding use, concerns have persisted regarding a potential association between GLP-1 receptor agonists (GLP-1 RAs) and pancreatitis, raising questions about the pancreatic safety of this drug class.

The controversy surrounding GLP-1 RAs and pancreatitis arises from conflicting evidence in the literature. Early pharmacovigilance analyses and observational studies reported increased rates of acute pancreatitis among GLP-1 RA users, particularly with exenatide, and suggested a possible association with pancreatic injury [2,3]. Proposed mechanisms included incretin-mediated pancreatic ductal proliferation and exocrine dysplasia based on preclinical and limited human data [4,5]. In parallel, randomized-trial data and meta-analyses have demonstrated an increased risk of gallbladder and biliary disease with GLP-1 RA use, raising the possibility that any observed pancreatitis signal may be mediated through biliary rather than intrinsic pancreatic mechanisms [6].

More recent large observational studies and systematic reviews, however, have not demonstrated a consistent association between GLP-1 RA use and acute pancreatitis [7,8,9,10]. A recent U.S.-based TriNetX cohort study by Eldesouki et al. [11] provided important additional context. At three years, GLP-1 RA use was associated with modestly higher odds of cholelithiasis or choledocholithiasis (adjusted odds ratio [aOR] 1.43, 95% CI 1.24–1.63), cholecystitis (aOR 1.45, 95% CI 1.14–1.83), and cholecystectomy (aOR 1.54, 95% CI 1.17–2.02), but not acute pancreatitis (aOR 0.82, 95% CI 0.63–1.04) or ERCP [11]. The biliary associations varied by agent, with significant signals for semaglutide and dulaglutide but not liraglutide or exenatide. These findings reinforce a biliary safety signal while providing no evidence of increased acute pancreatitis. Nevertheless, that study used a broad non-GLP-1 RA oral antidiabetic comparison group rather than a prespecified active-comparator class, was limited to three years of follow-up, and did not evaluate downstream pancreatic outcomes such as chronic pancreatitis, pancreatic pseudocyst, or pancreatic cancer.

Acute pancreatitis is associated with substantial morbidity and can lead to chronic pancreatitis, pancreatic pseudocyst formation, and, over time, pancreatic malignancy [12,13]. Given the widespread and often long-term use of GLP-1 RAs, a comprehensive evaluation of both acute pancreatitis and longer-term pancreatic outcomes remains clinically important. We therefore used a large global electronic health record network and an active-comparator, propensity-matched new-user design to evaluate pancreatic outcomes following GLP-1 RA initiation in adults with T2DM. GLP-1 RA initiators were compared with patients initiating SGLT2 inhibitors and, in sensitivity analyses, other second-line diabetes therapies. Outcomes included overall and biliary acute pancreatitis, chronic pancreatitis, pancreatic pseudocyst, pancreatic procedures, and pancreatic cancer over follow-ups extending to 10 years. By combining clinically relevant active comparators, a global cohort of more than 300,000 matched patients, and evaluation across the spectrum of acute and long-term pancreatic outcomes, this study extends prior work beyond short-term pancreatitis and gallbladder or biliary endpoints.

## 2. Materials and Methods

### 2.1. Data Source and Study Design

We conducted a retrospective, propensity-matched cohort study using the TriNetX platform, a global federated network of de-identified electronic health records from approximately 170 health care organizations across 21 countries, comprising over 190 million patients at the time of analysis. TriNetX enables patient-level analyses while maintaining anonymity by reporting aggregate results; counts between 1 and 10 are rounded to further preserve privacy. The MetroHealth Medical Center Institutional Review Board determined that studies using TriNetX are exempt from review because the data are de-identified and aggregated, in accordance with Section §164.514(a) of the HIPAA Privacy Rule.

### 2.2. Patient Selection and Cohort Construction

We identified adults aged ≥18 years who were alive at the index date with an active diagnosis of T2DM in the TriNetX network, between 1 January 2013 and 31 December 2025. The index event was the initiation of a GLP-1 RA for the primary cohort. The active-comparator cohort consisted of patients initiating a sodium–glucose cotransporter-2 inhibitor (SGLT2i). Diagnostic, procedural, and medication codes used to define cohorts are provided in Supplementary Table S3.

SGLT2 inhibitors were selected as the primary comparator because they are prescribed in a clinically similar population and have not been independently associated with pancreatitis after accounting for glycemic and weight-related effects [1]. To ensure clinical equipoise given differences in market availability, patients were excluded if the initiation of GLP-1 RAs, SGLT2 inhibitors, or comparator therapies occurred before 2013 [14].

As a prespecified sensitivity analysis, a secondary comparator cohort was constructed, comprising patients initiating other second-line diabetes therapies, specifically thiazolidinediones and sulfonylureas. This combined cohort was used only as a secondary sensitivity comparator to assess the robustness of the primary GLP-1 RA versus SGLT2i comparison and was not intended as the primary causal contrast. Dipeptidyl peptidase-4 (DPP-4) inhibitors were not included because of their shared incretin-based mechanism with GLP-1 RAs.

### 2.3. Exclusion Criteria

Patients were excluded if they had conditions known to strongly predispose to pancreatitis, including serum triglyceride levels >500 mg/dL [15], cystic fibrosis, primary sclerosing cholangitis, prior bariatric surgery, congenital pancreatic or biliary anomalies, alcohol use disorder, or alcohol-related acute or chronic pancreatitis.

To minimize confounding from drug-induced pancreatitis, patients receiving medications with a definite association with acute pancreatitis (Class 1a), including 5-aminosalicylates, arsenic trioxide, carbimazole, cimetidine, isoniazid, α-methyldopa, nadolol, perindopril, procainamide, sulindac, tamoxifen, and telaprevir, were excluded [16]. Each of these medications individually accounted for <0.1% of eligible patients.

Additional exclusions included any history of malignant or neuroendocrine neoplasms, prior acute or chronic pancreatitis, pancreatobiliary diagnoses or procedures that could preclude attribution of drug- or biliary-related pancreatitis (e.g., cholecystectomy, ERCP, pancreatic resection), serum IgG4 levels >280 mg/dL [17], or other biliary diseases as defined by MedDRA version 22.0 [18], before the index event (Supplementary Table S3).

Patients with prior pancreatobiliary procedures or conditions that would substantially interfere with attribution of incident pancreatitis were excluded; uncomplicated cholelithiasis was not excluded and was retained as a propensity score covariate because of its established association with biliary pancreatitis.

Patients initiating GLP-1 RAs had no prior or concurrent exposure to SGLT2 inhibitors, and vice versa. The same mutual exclusivity criteria were applied in sensitivity analyses comparing GLP-1 RAs with other second-line diabetes therapies.

### 2.4. Covariates

**Figure 1:**
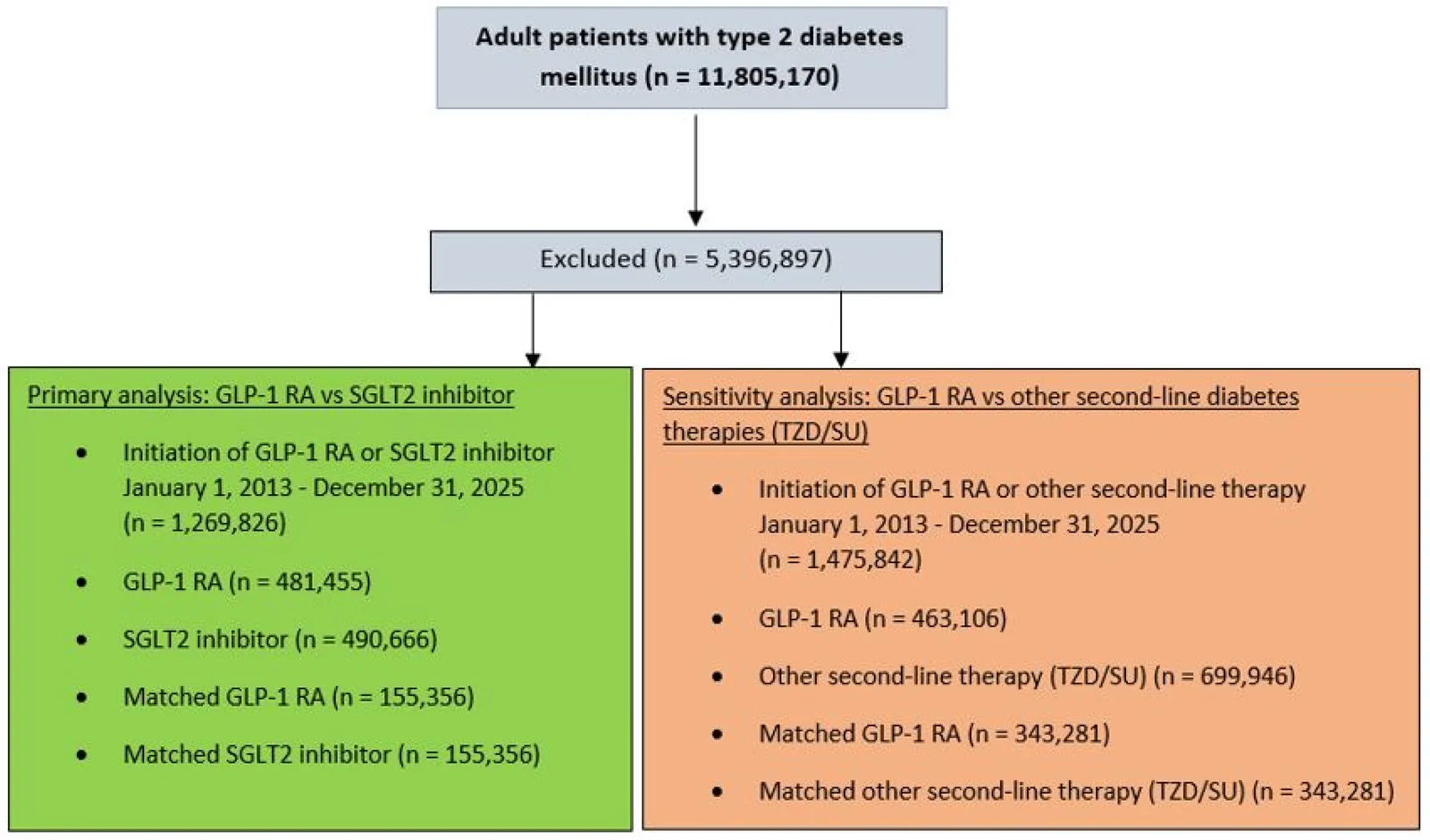
Flow diagram of participant selection and cohort construction. The primary analysis compared initiation of glucagon-like peptide-1 receptor agonists (GLP-1 RAs) with sodium–glucose cotransporter-2 inhibitors (SGLT2i). Sensitivity analyses compared GLP-1 RAs with other second-line diabetes therapies (thiazolidinediones [TZD] or sulfonylureas [SU]).

We used the American Diabetes Association’s 2022 T2DM management guidelines [1] to identify confounding factors predicting GLP-1 RA versus SGLT2i initiation (Figure 1), as documented by diagnostic codes (Supplementary Table S3). We also identified confounding factors that could predict the risk of pancreatic outcomes, including initial A1c, initial BMI, prior history of cholelithiasis, hypercalcemia, nicotine dependence, use of opioids, octreotide, ceftriaxone, diabetic complications, or fibrates. Pregnancy was also identified by choriogonadotropin presence of >25 m[IU]/mL or by diagnostic codes (Supplementary Table S3). Commonly used Class 1a pancreatitis-associated medications (>0.1% prevalence) were identified and included as covariates in cohort balancing. These included acetaminophen, acetaminophen-codeine, amiodarone, androgenic anabolic steroids, cannabis, enalapril, estrogen and related products, furosemide, losartan, metronidazole, pravastatin, pyritinol, ranitidine, rosuvastatin, DPP-4 inhibitors, simvastatin, tetracycline, and trimethoprim/sulfamethoxazole. Propensity score matching was performed between the GLP-1 RA and SGLT2i cohorts, incorporating demographic variables, comorbidities, and established risk factors for pancreatitis, including obesity-related measures, gallstone disease, hypertriglyceridemia, relevant medication exposures, and indications for specific T2DM therapies, as well as age at index event, sex, ethnicity, and race [19].

### 2.5. Study Outcomes

The primary outcome was incident acute pancreatitis, defined using ICD-10-CM K85 as a first-ever diagnosis recorded during an inpatient or emergency-care encounter occurring at least 1 day after the index event. Prespecified etiology-specific analyses were performed for biliary acute pancreatitis. Secondary outcomes included incident chronic pancreatitis and complications of pancreatitis, including pancreatic pseudocyst. Pancreatic cancer was evaluated as an additional outcome and was defined as a first-ever diagnosis of malignant neoplasm of the pancreas (ICD-10-CM C25) occurring after the index event. As an exploratory measure of downstream pancreatobiliary diagnostic and interventional utilization, we assessed the first-ever receipt of a composite of endoscopic retrograde cholangiopancreatography (ERCP), endoscopic ultrasound (EUS), or magnetic resonance cholangiopancreatography (MRCP). These procedures have distinct clinical indications and were not considered direct measures of pancreatic injury or pancreatic complications.

Outcomes were identified using diagnosis and procedure codes recorded during inpatient or emergency encounters to enhance specificity for clinically significant events. Patients were followed from treatment initiation for up to 10 years, until outcome occurrence, death, loss to follow-up, or end of data availability. The same outcome definitions were applied across all sensitivity analyses.

### 2.6. Sensitivity Analyses

To assess the robustness of the primary findings, we performed several prespecified sensitivity analyses. First, we repeated all analyses in a USA-only cohort, restricting the study population to patients receiving care within U.S.-based health care organizations. The same inclusion and exclusion criteria, index event definitions, outcome definitions, and propensity score matching procedures used in the primary analysis were applied to this cohort.

Second, to evaluate whether observed associations were dependent on the choice of comparator, we conducted an additional sensitivity analysis comparing patients initiating GLP-1 receptor agonists with patients initiating second-line diabetes medications other than SGLT2 inhibitors. These comparator therapies included commonly used non-incretin, non-SGLT2 second-line agents and excluded dipeptidyl peptidase-4 inhibitors due to shared incretin-based mechanisms. As in the primary analysis, cohorts were mutually exclusive, and patients had no prior exposure to the comparator drug class before the index event. Time-horizon sensitivity analysis analysis was performed for any non-proportional models at 1 year, 3 years and 10 years. To address potential reverse causation and detection bias for pancreatic cancer, additional sensitivity analyses were performed using 6- and 12-month lag periods, excluding pancreatic cancer events occurring during the respective lag intervals.

### 2.7. Statistical Analysis

All statistical analyses were conducted on the TriNetX platform in real time [20]. As appropriate, outcomes were described using means, standard deviations (SD), and proportions. One-to-one propensity score matching was performed using greedy nearest-neighbor algorithms with a caliper width of 0.1 pooled standard deviations based on relevant covariates. Characteristics with a standardized mean difference between cohorts of less than 0.1 were considered well-matched [21]. We performed Kaplan–Meier analysis on the propensity-matched groups as described by Austin et al., 2014 [22]. Follow-up began at initiation of the index therapy and continued until outcome occurrence, death, loss to follow-up, or end of data availability. We censored patients after their last data point on record or when deceased. We performed log-rank testing on the curves to assess significant differences. We used the Benjamini & Hochberg method to correct for multiple testing since outcome variables may be related to each other [23]. A *p*-value < 0.05 was considered significant after correction. Hazard ratios (HR) were calculated using a univariate Cox proportional hazard model with 95% confidence intervals. TriNetX tested for proportionality using the scaled Schoenfeld residual. Figures and analysis were generated in Microsoft Excel (Redmond, WA, USA) version 2208 and R version 4.4.2 (Vienna, Austria) [24]. Detailed baseline characteristics before and after propensity score matching are provided in Supplementary Tables S1 and S2, the diagnostic, procedural, and medication codes used for cohort construction, propensity score matching, and outcome definitions are provided in Supplementary Table S3, and additional methodological details are provided in the Supplemental Methods. Prespecified sensitivity analyses included comparisons with alternative second-line T2DM therapies and restriction to patients receiving care within the United States.

This study was reported in accordance with the Strengthening the Reporting of Observational Studies in Epidemiology (STROBE) statement. Completed checklists are provided in Supplementary Table S5.

## 3. Results

From a source population of 190,888,407 patients [20], we identified 11,805,170 adults with T2DM, of whom 5,396,897 met the predefined exclusion criteria. Among the eligible population, 463,106 patients initiated a GLP-1 RA and 490,666 initiated a SGLT2i. After 1:1 propensity score matching, 291,152 patients were included in each cohort. Baseline characteristics were generally well balanced after propensity score matching. Although residual imbalance remained for selected variables, including mean BMI, BMI category distributions were well balanced, with standardized mean differences <0.1 across BMI strata (Supplementary Tables S1 and S2).

**Figure 2:**
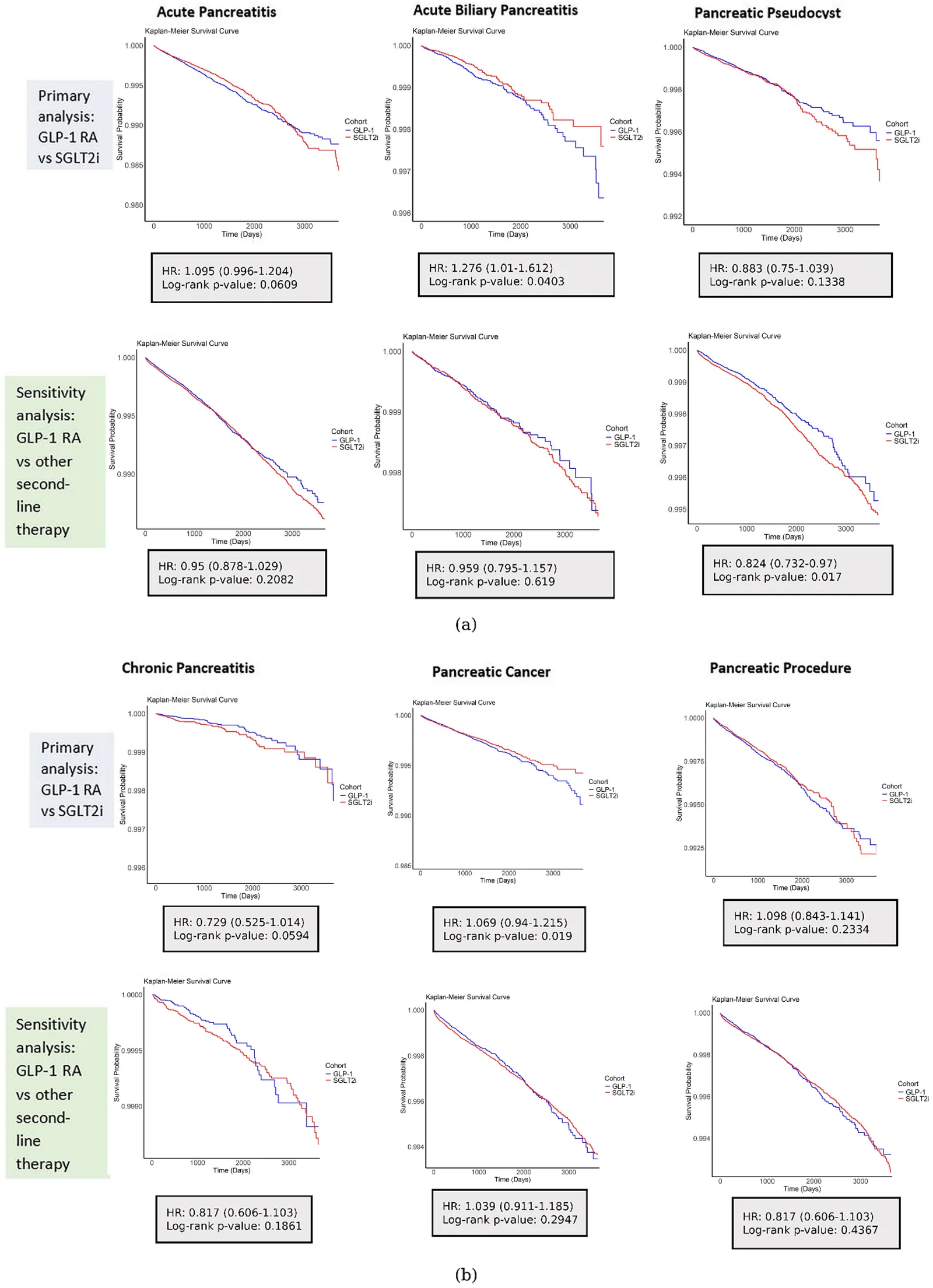
Kaplan–Meier curves for pancreatitis and pancreatitis-related outcomes in global patients with type 2 diabetes mellitus initiating glucagon-like peptide-1 receptor agonists (GLP-1 RAs) compared with sodium–glucose cotransporter-2 inhibitors (SGLT2i) or other second-line diabetes therapies. The top row shows the primary comparison between GLP-1 RAs and SGLT2 inhibitors. The bottom row shows sensitivity analyses comparing GLP-1 RAs with other second-line therapies (thiazolidinediones or sulfonylureas). Hazard ratios with 95% confidence intervals and log-rank *p*-values are shown for each outcome.

**Figure 3:**
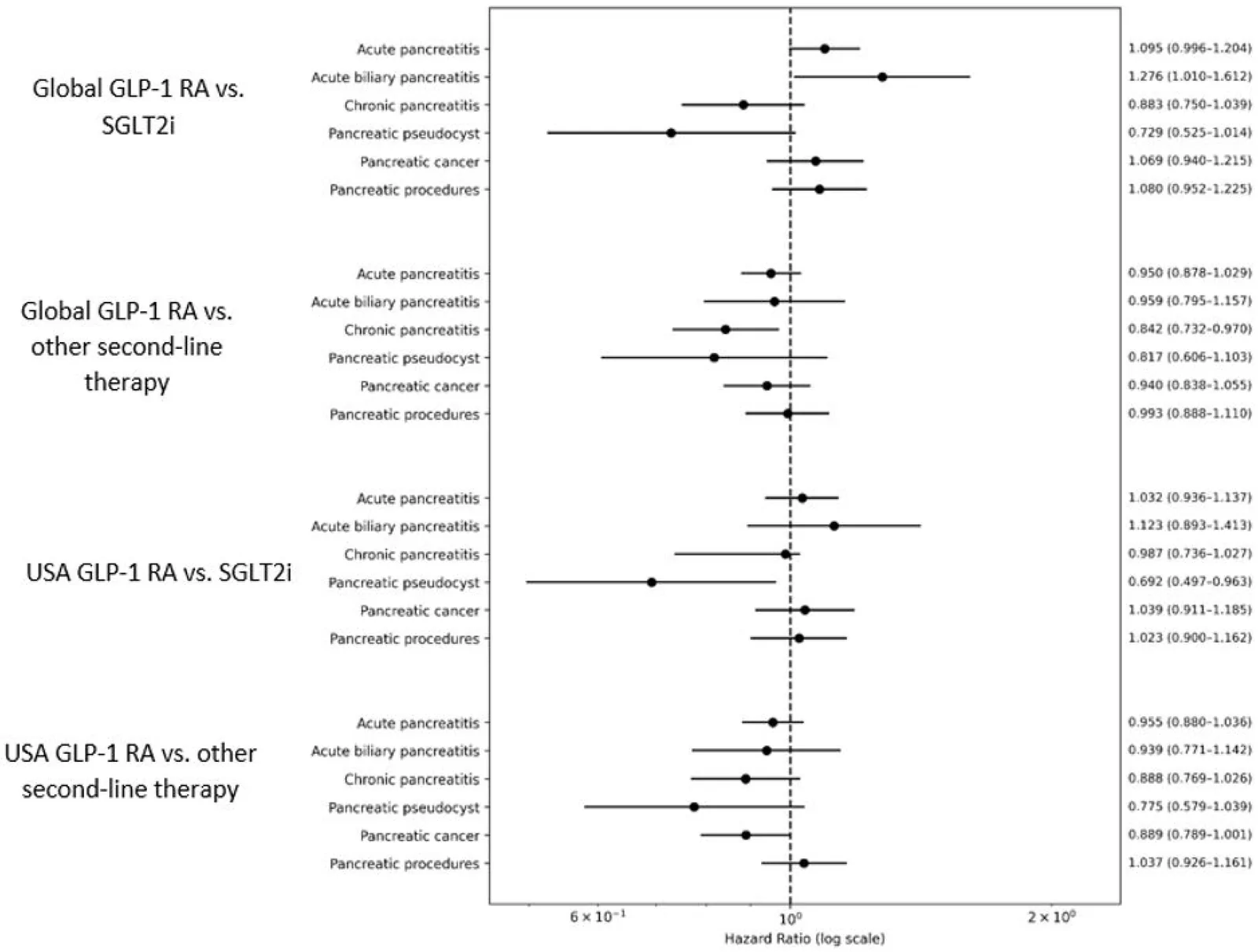
Cox proportional hazards ratios for pancreatic outcomes following initiation of glucagon-like peptide-1 receptor agonists (GLP-1 RAs) compared with sodium–glucose cotransporter-2 inhibitors (SGLT2i) or other second-line diabetes therapies. Hazard ratios with 95% confidence intervals are shown for the primary global comparison, sensitivity analyses using alternative second-line comparators, and analyses restricted to U.S.-based patients. The vertical dashed line indicates a hazard ratio of 1.0.

Beginning 1 day after the index event, 953 patients in the GLP-1 RA cohort developed overall acute pancreatitis compared with 772 patients in the SGLT2i cohort, corresponding to cumulative incidences of 0.33% and 0.27%, respectively. Kaplan–Meier analyses demonstrated no significant difference in cumulative incidence between cohorts, which was confirmed by log-rank testing before and after correction for multiple testing (Figure 2). Consistent with these findings, time-to-event analyses showed no difference in the hazard of overall acute pancreatitis between GLP-1 RA and SGLT2i initiation (HR 1.095; 95% CI 0.996–1.204; Figure 3).

In an etiology-specific subanalysis, GLP-1 RA initiation was associated with a higher hazard of biliary acute pancreatitis compared with SGLT2i initiation (HR 1.276; 95% CI 1.01–1.612); this association was not consistently observed across sensitivity analyses. No difference was observed in the risk of chronic pancreatitis between the two cohorts (HR 0.883; 95% CI 0.75–1.039).

When evaluating pancreatitis-related complications, there was no difference in the incidence of pancreatic pseudocysts (HR 0.729; 95% CI 0.525–1.014). In the exploratory analysis of pancreatobiliary diagnostic and interventional utilization, the composite incidence of MRCP, EUS, or ERCP did not differ between cohorts (HR 1.080, 95% CI 0.952–1.225).

For pancreatic cancer, the overall hazard did not differ between GLP-1 RA and SGLT2i initiation (HR 1.069; 95% CI 0.94–1.215). Time-horizon sensitivity analysis analyses demonstrated no increased risk of pancreatic cancer at 1, 3, or 10 years following treatment initiation (Supplementary Table S4). Hazard ratios at 1 year (HR 0.954; 95% CI 0.771–1.181) and 10 years (HR 1.06; 95% CI 0.93–1.20) remained non-significant, although a modest upward trend over time was observed. To address potential reverse causation and detection bias, additional lagged sensitivity analyses were performed. The association remained non-significant after a 6-month lag (HR 1.118, 95% CI 0.953–1.311; log-rank *p* = 0.1715), 12-month lag (HR 1.138, 95% CI 0.952–1.361; log-rank *p* = 0.1565) and 24-month lag (HR: 1.073, 95% CI 0.878–1.312 Log-Rank *p* = 0.4899).

### Sensitivity Analysis

In analyses restricted to patients receiving care within the United States, no differences were observed between GLP-1 RA and SGLT2i initiation for acute pancreatitis (HR 1.032; 95% CI 0.936–1.137), biliary acute pancreatitis (HR 1.123; 95% CI 0.893–1.413), or chronic pancreatitis (HR 0.987; 95% CI 0.736–1.027; Figure 3).

Similarly, when GLP-1 RA initiators were compared with patients initiating other second-line diabetes medications (excluding SGLT2 inhibitors), no differences were observed for acute pancreatitis (HR 0.95; 95% CI 0.878–1.029) and biliary acute pancreatitis (HR 0.959; 95% CI 0.795–1.157). Although chronic pancreatitis was associated with a lower hazard in the GLP-1 RA cohort compared with other second-line therapies (HR 0.842; 95% CI 0.732–0.970), this association was not consistently observed across the other comparator and sensitivity analyses.

Evaluation of longer-term pancreatic outcomes in the U.S. cohort demonstrated no difference in pancreatic cancer risk (HR 1.039; 95% CI 0.911–1.185). Although pancreatic pseudocyst formation was associated with a lower hazard in GLP-1 RA initiators compared with SGLT2i initiators (HR 0.692; 95% CI 0.497–0.963), this finding was not consistently observed across other comparator and sensitivity analyses. This lower pseudocyst hazard was not reproduced when GLP-1 RA initiators were compared with other second-line diabetes therapies (Figure 3).

## 4. Discussion

This study represents the large real-world, population-based analysis to date, examining the association between GLP-1 RA use and pancreatitis and related pancreatic outcomes. In this large electronic medical record-based study [20], initiation of GLP-1 RAs in adults with T2DM was not associated with an increased risk of incident acute pancreatitis, chronic pancreatitis, pancreatic procedures, or pancreatic cancer compared with the initiation of SGLT2i or other second-line diabetes therapies. These findings were consistent across multiple prespecified sensitivity analyses and extended follow-up periods of up to 10 years.

The relationship between GLP-1 RAs and acute pancreatitis has been controversial. Early observational studies and pharmacovigilance reports suggested a possible increased risk, particularly within the first months to years following treatment initiation [2,3,25]. Subsequent large cardiovascular outcome trials of sitagliptin, saxagliptin, and alogliptin did not individually demonstrate a statistically significant increase in acute pancreatitis. However, pooled analyses have yielded mixed results, with some meta-analyses of DPP-4 inhibitor cardiovascular outcome trials suggesting a small increase in relative risk, whereas other broader randomized-trial meta-analyses have not confirmed this association. More recent observational studies and meta-analyses have not demonstrated a consistent, clinically meaningful association between GLP-1 RA use and acute pancreatitis [10,26,27]. Our findings strengthen this body of evidence.

These results are concordant with those of Eldesouki et al. (2026) [11], who similarly reported no significant association between GLP-1 RA use and acute pancreatitis at either two years, despite increased gallbladder and biliary events [11]. Our study extended these observations using prespecified active comparators within a large global network, longer follow-up, and evaluations of chronic pancreatitis, pancreatic pseudocyst, pancreatic procedures, and pancreatic cancer.

A modest association in biliary acute pancreatitis was observed in the primary comparison with SGLT2 inhibitors. This finding is biologically plausible given the effects of GLP-1 RAs on gallbladder motility and weight loss, both of which may increase the risk of gallstone formation and biliary complications. Importantly, this association was not consistently reproduced across sensitivity analyses and was not accompanied by increased overall acute pancreatitis, chronic pancreatitis, pancreatic pseudocyst, or pancreatic cancer, arguing against a clear signal of reflecting intrinsic pancreatic toxicity.

Overall, pancreatitis and pancreatic pseudocyst formation did not show consistent association with GLP-1 RA initiation across comparator and sensitivity analyses. Isolated lower-hazard signals were observed for chronic pancreatitis with other second-line therapies and for pancreatic pseudocyst formation in the U.S. SGLT2i comparison, but neither finding was consistently reproduced across analyses. These comparator-specific findings may reflect residual confounding rather than a protected effect of GLP-1 RAs.

Prior studies evaluating long-term pancreatic outcomes have been limited by relatively short follow-up durations and small cohort sizes. For example, Knapen et al. reported numerically higher incidences of pancreatitis with liraglutide based on a small number of events [28]. Earlier case–control studies suggested increased rates of pancreatic or biliary malignancy among GLP-1 RA users, but follow-up in these analyses was typically limited to two to three years [2,3], which is likely insufficient to evaluate pancreatic carcinogenesis given its long latency. Expert opinion suggests that a minimum of 5–6 years of follow-up is required to meaningfully assess pancreatic cancer risk [29]. In contrast, our study benefits from an extended follow-up of up to 10 years, time-to-event analyses accounting for variable follow-up, and large matched cohorts exceeding 150,000 patients per group, allowing for more reliable evaluations of rare outcomes such as pancreatic cancer. Our findings are consistent with prior meta-analyses demonstrating no increased pancreatic cancer risk associated with GLP-1 RA use [30,31]. Although the proportional hazards assumption was violated for pancreatic cancer, the overall hazard ratio was not significant, and time-horizon sensitivity analysis analyses showed no increased risk over time.

Several methodological differences between the present study and Eldesouki et al. should be noted. Eldesouki et al. [11] excluded insulin-treated patients and required at least two documented GLP-1 RA prescriptions, whereas the present study defined exposure at treatment initiation and applied more extensive exclusions and propensity matching for pancreatitis-related risk factors. In addition, Eldesouki et al. primarily reported adjusted odds ratios, whereas the present study used Cox proportional hazards models and Kaplan–Meier analyses to account for variable follow-up. Despite these differences, both studies found no significant association between GLP-1 RA use and acute pancreatitis.

Several limitations should be acknowledged. First, GLP-1 RAs and SGLT2 inhibitors entered the market at different times. To mitigate potential bias, analyses were restricted to periods of overlapping drug availability beginning in 2013, and agents withdrawn from the market were excluded. In contrast, the Eldesouki et al. study included patients from 2007 onward, reflecting the earlier FDA approval of exenatide, and used a broader inclusion window that may have introduced temporal confounding related to evolving prescribing patterns and coding practices. Second, as with all diagnostic code-based observational studies, outcome misclassification and residual confounding cannot be fully excluded, and causal inference is limited. Acute pancreatitis was ascertained using diagnosis codes rather than adjudicated clinical criteria, and some outcome misclassification remains possible. Restricting acute pancreatitis diagnoses to inpatient or emergency-care encounters was intended to improve specificity; however, this coding definition should not be considered equivalent to a prospectively validated clinical diagnosis incorporating symptoms, imaging, and pancreatic enzyme measurements. However, stringent exclusion criteria, propensity score matching, and active-comparator designs were used to minimize confounding related to disease severity and treatment selection. The Eldesouki et al. study similarly acknowledged that the TriNetX platform does not reliably capture medication discontinuation dates, treatment duration, or switching between therapies, a limitation shared by the present study. Although follow-up extended to 10 years, only patients initiating therapy during the earlier years of the study period could contribute to the longest follow-up intervals. Detailed risk-set distributions and agent-specific compositions of the longest-followed patients were not available from the aggregate TriNetX outputs. Accordingly, the 10-year findings should be interpreted cautiously and should not be directly extrapolated to newer agents, including semaglutide and tirzepatide, for which substantially shorter longitudinal follow-up is available. Accordingly, these long-term findings should not be directly extrapolated to newer agents, including semaglutide and tirzepatide, for which substantially shorter longitudinal follow-up is currently available. In addition, the secondary comparator cohort combined sulfonylureas and thiazolidinediones, which represent pharmacologically and clinically heterogeneous treatment classes. Although this cohort was included only as a sensitivity comparator and not as the primary causal contrast, such heterogeneity may contribute to residual confounding and should be considered when interpreting these secondary analyses. Finally, medication dose and adherence could not be assessed within the TriNetX platform; future studies should evaluate dose–response relationships and agent-specific effects. Agent-specific analyses, such as those performed by Eldesouki et al. demonstrating heterogeneity in biliary risk across semaglutide, dulaglutide, liraglutide, and exenatide, represent an important direction for future research on pancreatic outcomes as well.

## 5. Conclusions

In conclusion, in this large global propensity-matched cohort study with extended longitudinal follow-up, initiation of GLP-1 receptor agonists was not associated with an increased risk of acute pancreatitis, pancreatitis-related complications, or pancreatic cancer compared with SGLT2 inhibitors or other second-line diabetes therapies in adults with T2DM. These findings provide reassurance regarding the pancreatic safety of GLP-1 RAs in routine clinical practice. Future prospective studies with more granular exposure data may further refine risk estimates in specific high-risk populations.

## Data Availability

The data used in this study were obtained from the TriNetX research network. Because TriNetX provides access to de-identified, aggregated electronic health record data under data-use agreements, individual-level patient data are not publicly available. Data supporting the findings of this study may be available through TriNetX to qualified investigators with appropriate institutional access and approvals.

## Supplementary Materials

The following supporting information can be downloaded at: https://www.mdpi.com/article/10.3390/jpm16090478/s1. Table S1: Baseline characteristics of patients initiating glucagon-like peptide-1 receptor agonists and sodium-glucose cotransporter-2 inhibitors before and after propensity score matching; Table S2: Baseline characteristics of patients initiating glucagon-like peptide-1 receptor agonists and other second-line diabetes therapies before and after propensity score matching; Table S3: Diagnostic, Procedure, and Medication Codes Used for Query, Propensity Matching, or Outcomes; Table S4: Time-specific analyses of pancreatic cancer following GLP-1 RA versus SGLT2i initiation; Table S5. STROBE Statement—Checklist for Cohort Studies. Supplemental Methods.
