# Supplementary material for: Glucagon-like Peptide-1 Receptor Agonists Are Not Associated with Increased Risk of Acute Pancreatitis or Pancreatic Complications, Including Pancreatic Cancer, in Type 2 Diabetes: A Global Propensity-Matched Cohort Study
Source: J Pers Med. 2026 Sep 16;16(9):478. doi: 10.3390/jpm16090478 (PMC13608214; doi:10.3390/jpm16090478)
Supplement: Supplementary file 1 [file jpm-16-00478-s001.zip › jpm-4427954-supplementary.pdf]

Supplementary Table S1: Baseline characteristics of patients initiating glucagon-like peptide-1 receptor agonists and sodium-glucose cotransporter-2 inhibitors before and after propensity score matching.

	After match Cohort, No. (%)			Before match Cohort, No. (%)		
Std Diff.	Control	GLP-1 RA	Std Diff.	Control	GLP-1 RA	**Characteristic**
	291,152	291,152		490,666	463,106	Total No.
0.036	60.5 +/- 12.7	60 +/- 12.3	0.522	63 +/- 12.8	56.1 +/- 13.6	Age, mean (SD), y
						Sex
0.005	141,505 (48.6)	142,286 (48.9)	0.412	193,187 (39.4)	275,787 (59.5)	Female (%)
0.005	149,363 (51.3)	148,571 (51.0)	0.412	297,052 (60.5)	186,919 (40.4)	Male (%)
						Ethnicity
0.008	27,150 (9.3)	26,494 (9.1)	0.06	37,877 (7.7)	43,618 (9.4)	Hispanic/LatinX(%)
0.037	194,066 (66.7)	189,027 (64.9)	0.216	284,815 (58.1)	316,760 (68.4)	Not Hispanic/LatinX(%)
0.045	69,936 (24)	75,631 (26)	0.27	167,974 (34.2)	102,728 (22.2)	Unknown(%)
						Race
0.001	14,116 (4.9)	14,147 (4.9)	0.178	37,043 (7.6)	16,186 (3.5)	Asian(%)
0.011	56,045 (19.3)	54,794 (18.8)	0.165	73,998 (15.1)	99,336 (21.5)	Black(%)
0.03	170,878 (58.7)	166,571 (57.2)	0.265	232,911 (47.5)	280,532 (60.6)	White(%)
0.108	11,983 (4.1)	19,044 (6.5)	0.011	24,118 (4.9)	23,867 (5.2)	Other(%)
						Characteristics
0.01	8.0 (2.0)	8.0 (2.1)	0.042	7.9 (1.9)	7.8 (2.1)	Initial A1c mean (SD), %
0.25	33.1 (7.7)	35 (7.9)	0.692	31.6 (7.4)	37.1 (8.4)	Initial BMI mean (SD), kg/m2
0.015	71,224 (30.3)	69,538 (29.6)		104,398 (23.89)	116,197 (31.9)	BMI 30-34.9 (%)
0.0009	52,843 (22.50)	52,753 (22.46)		65,198 (15.0)	111,300 (30.5)	BMI 35-40
0.0043	39,440 (16.8)	39,822 (16.9)		44,261 (10.1%)	104,885 (28.76)	BMI >40
						Comorbidities
0.004	134,407 (46.2)	133,849 (46)	0.082	233,615 (47.6)	201,555 (43.5)	Hyperlipidemia(%)
0.016	188,114 (64.6)	185,885 (63.9)	0.014	312,032 (63.6)	291,361 (62.9)	Primary Hypertension(%)
0.01	14,697 (5.1)	14,108 (4.9)	0.1	29,463 (6.1)	17,789 (3.8)	Peripheral vascular disease(%)
0.017	53,261 (18.3)	51,360 (17.6)	0.361	131,009 (26.7)	58,243 (12.6)	Coronary artery disease(%)

0.009	14,001 (4.8)	13,474 (4.6)	0.124	30,665 (6.3)	16,524 (3.6)	Atherosclerosis(%)
0.008	13,712 (4.7)	13,209 (4.5)	0.133	31,351 (6.4)	16,279 (3.5)	Cerebral infarction(%)
0.003	5,591 (1.9)	5,459 (1.9)	0.006	10,117 (2.1)	9,159 (2)	Cholelithiasis(%)
0.019	44,163 (15.2)	42,242 (14.5)	0.22	93,608 (19.1)	51,977 (11.2)	Chronic Kidney Disease(%)
0.004	40,522 (13.9)	40,172 (13.8)	0.022	65,633 (13.4)	58,584 (12.7)	Diabetes with Neurologic complication (%)
0.008	20,092 (6.9)	19,499 (6.7)	0.079	37,571 (7.7)	26,383 (5.7)	Diabetes with Circulatory complications(%)
0.001	9,554 (3.3)	9,593 (3.3)	0.007	14,614 (3)	14,338 (3.1)	Unspecified diabetic retinopathy(%)
0.001	6,814 (2.3)	6,782 (2.3)	0.013	10,004 (2)	10,325 (2.2)	Mild nonproliferative diabetic retinopathy(%)
0.001	2,398 (0.8)	2,375 (0.8)	0.008	3,456 (0.7)	3,583 (0.8)	Moderate nonproliferative diabetic retinopathy (%)
0.001	1,088 (0.4)	1,076 (0.4)	0.001	1,633 (0.3)	1,524 (0.3)	Severe nonproliferative diabetic retinopathy(%)
0.001	3,853 (1.3)	3,814 (1.3)	0.006	6,270 (1.3)	5,536 (1.2)	proliferative diabetic retinopathy(%)
0.008	34,161 (11.7)	33,409 (11.5)	0.024	55,045 (11.2)	55,523 (12)	Nicotine dependence(%)
						Other States or Conditions
0.001	1,484 (0.5)	1,667 (0.6)	0.146	1,543 (0.3)	8,339 (1.8)	Pregnancy(%)
						Common Class 1a medications
0.001	3,003 (1)	3,029 (1)	0.012	5,030 (1)	5,297 (1.1)	Cannabis-related disorder(%)
0.003	315 (0.1)	282 (0.1)	0.017	639 (0.1)	345 (0.1)	Octreotide(%)
0.006	22,947 (7.9)	22,453 (7.7)	0.056	44,414 (9.1)	34,795 (7.5)	Ceftriaxone(%)
0.014	118,962 (40.9)	116,952 (40.2)	0.062	195,429 (39.8)	198,630 (42.9)	Opioids(%)
0.002	12,193 (4.2)	12,108 (4.2)	0.027	20,901 (4.3)	17,312 (3.7)	Fibrates(%)
0.009	132,136 (45.4)	130,871 (45)	0.006	230,419 (47)	218,812 (47.3)	Acetaminophen(%)
0.001	23,213 (8)	23,152 (8)	0.137	32,394 (6.6)	48,266 (10.4)	Codeine(%)
0.016	6,755 (2.3)	6,056 (2.1)	0.213	25,028 (5.1)	6,291 (1.4)	Amiodarone
0.001	130 (0.1)	136 (0.1)	0.018	163 (<0.1)	346 (0.1)	Anabolic Steroids(%)
0.004	5,445 (1.9)	5,284 (1.8)	0.019	9,541 (1.9)	7,856 (1.7)	Enalapril(%)
0.01	10,958 (3.8)	11,491 (4)	0.248	14,485 (3)	40,411 (8.7)	Systemic hormonal contraceptives(%)
0.008	9,904 (3.4)	10,350 (3.6)	0.251	10,974 (2.2)	35,302 (7.6)	Estrogens(%)

0.018	50,159 (17.3)	48,212 (16.6)	0.243	111,260 (22.7)	62,061 (13.4)	Furosemide(%)
0.011	54,411 (18.7)	53,145 (18.3)	0.008	89,252 (18.2)	82,800 (17.9)	Losartan(%)
0.002	19,841 (6.8)	19,955 (6.9)	0.15	28,520 (5.8)	45,534 (9.8)	Metronidazole(%)
<0.001	5,240 (1.8)	5,244 (1.8)	0.017	9,432 (1.9)	10,031 (2.2)	Pyridoxine(%)
0.002	11,967 (4.1)	11,853 (4.1)	0.079	17,231 (3.5)	23,687 (5.1)	Ranitidine (%)
0.003	38,468 (13.2)	38,161 (13.1)	0.058	69,027 (14.1)	56,154 (12.1)	Rosuvastatin (%)
0.098	44,183 (15.2)	34,487 (11.9)	0.178	82,544 (16.8)	49,658 (10.7)	Dipeptidyl Peptidase-4 inhibitors(%)
0.005	28,091 (9.7)	27,650 (9.5)	0.004	44,869 (9.2)	41,760 (9)	Simvastatin(%)
0.003	35,388 (12.2)	35,124 (12.1)	0.127	52,351 (10.7)	69,035 (14.9)	Tetracyclines(%)
0.001	30,561 (10.5)	30,613 (10.5)	0.179	40,791 (8.3)	64,502 (13.9)	Trimethoprim(%)
0.001	28,056 (9.6)	28,099 (9.7)	0.161	38,461 (7.8)	58,883 (12.7)	Sulfamethoxazole(%)

Values are presented as mean (SD) or number (%), as appropriate. Standardized differences <0.1 indicate adequate balance between cohorts.

Supplementary Table S2: Baseline characteristics of patients initiating glucagon-like peptide-1 receptor agonists and other second-line diabetes therapies before and after propensity score matching.

**After Matching**			**Before Matching**			
		**Cohort, No. (%)**			**Cohort, No. (%)**	
Std Diff.	Control	GLP-1 RA	Std Diff.	Control	GLP-1 RA	**Characteristic**
	343,281	343,281		699,946	481,455	Total No.
0.033	58.9 +/- 13.2	58.5 +/- 12.3	0.509	62.4 +/- 12.9	55.7 +/- 13.4	Age, mean (SD), y
						Sex
0.001	177,492 (51.7)	177,298 (51.7)	0.277	306,166 (43.7)	276,663 (57.5)	Female(%)
0.001	165,553 (48.2)	165,684 (48.3)	0.277	393,339 (56.2)	204,421 (42.5)	Male(%)
						Ethnicity
0.012	34,791 (10.1)	33,524 (9.8)	0.033	61,181 (8.7)	46,745 (9.7)	Hispanic/LatinX(%)
0.029	230,130 (67)	225,384 (65.7)	0.25	395,618 (56.5)	329,863 (68.5)	Not Hispanic/LatinX(%)
0.041	78,360 (22.8)	84,373 (24.6)	0.291	243,147 (34.7)	104,847 (21.8)	Unknown(%)
						Race
0.005	14,580 (4.3)	14,951 (4.4)	0.126	44,289 (6.3)	17,284 (3.6)	Asian(%)

0.006	68,626 (20)	67,832 (19.8)	0.156	109,058 (15.6)	104,145 (21.6)	Black(%)
0.043	207,943 (60.6)	200,780 (58.5)	0.228	342,738 (49)	290,099 (60.3)	White(%)
0.113	12,990 (3.8)	21,474 (6.3)	0.029	33,139 (4.7)	25,807 (5.4)	Other(%)
						Characteristics
0.057	8.1 +/- 2.2	8.0 +/- 2.1	0.221	8.2 +/- 2.1	7.8 +/- 2.1	Initial A1c mean (SD), %
0.173	33.8 +/- 7.8	35.2 +/- 8.0	0.632	32.0 +/- 7.5	37 +/- 8.4	Initial BMI mean (SD), kg/m2
0.012	90,541 (27.8)	88,743 (27.3)	0.298	126,232 (18.4)	141,669 (31.2)	BMI 30-35 (%)
0.004	68,021 (20.9)	68,634 (21.1)	0.448	81,368 (11.9)	134,764 (29.6)	BMI 35-40
0.011	54,318 (16.7)	55,655 (17.1)	0.519	58,768 (8.59)	127,395 (28.0)	BMI>40
						Comorbidities
0.006	142,236 (41.4)	141,229 (41.1)	0.151	250,719 (35.8)	207,854 (43.2)	Hyperlipidemia(%)
0.006	209,941 (61.2)	206,013 (60)	0.172	378,309 (54.1)	300,804 (62.5)	Primary Hypertension(%)
0.006	14,441 (4.2)	14,016 (4.1)	<0.001	27,393 (3.9)	18,878 (3.9)	Peripheral vascular disease(%)
0.015	53,467 (15.6)	51,656 (15.1)	0.023	105,550 (15.1)	68,775 (14.3)	Coronary artery disease(%)
0.005	12,877 (3.8)	12,555 (3.7)	0.011	24,031 (3.4)	17,472 (3.6)	Atherosclerosis(%)
0.003	13,612 (4)	13,409 (3.9)	0.067	34,166 (4.9)	17,059 (3.5)	Stroke/TIA(%)
<0.001	5,451 (1.6)	5,437 (1.6)	0.053	8,947 (1.3)	9,331 (1.9)	Cholelithiasis(%)
0.015	42,855 (12.5)	41,196 (12)	0.027	85,850 (12.3)	54,925 (11.4)	Chronic Kidney Disease(%)
0.005	42,201 (12.3)	41,669 (12.1)	0.079	69,253 (9.9)	59,550 (12.4)	Diabetes with Neurologic complications(%)
0.004	18,688 (5.4)	18,404 (5.4)	0.081	28,815 (4.1)	28,301 (5.9)	Diabetes with Circulatory complications(%)
0.002	9,820 (2.9)	9,686 (2.8)	0.029	17,076 (2.4)	13,954 (2.9)	Unspecified diabetic retinopathy(%)
0.003	6,405 (1.9)	6,289 (1.8)	0.058	9,168 (1.3)	9,914 (2.1)	Mild nonproliferative diabetic retinopathy(%)
<0.001	2,279 (0.7)	2,267 (0.7)	0.043	3,030 (0.4)	3,696 (0.8)	Moderate nonproliferative diabetic retinopathy (%)
0.002	1,021 (0.3)	994 (0.3)	0.025	1,405 (0.2)	1,595 (0.3)	Severe nonproliferative diabetic retinopathy (%)
0.001	3,903 (1.1)	3,872 (1.1)	0.021	6,869 (1)	5,795 (1.2)	proliferative diabetic retinopathy(%)
0.006	37,291 (10.9)	36,703 (10.7)	0.127	57,880 (8.3)	58,295 (12.1)	Nicotine dependence(%)
						Other States or Conditions(%)

0.001	2,241 (0.7)	2,497 (0.7)	0.129	2,421 (0.4)	7,755 (1.6)	Pregnancy(%)
						Common Class 1a medications
0.004	2,843 (0.8)	2,955 (0.9)	0.072	3,681 (0.5)	5,746 (1.2)	Cannabis-related disorder(%)
<0.001	266 (0.1)	269 (0.1)	0.01	658 (0.1)	318 (0.1)	Octreotide(%)
0.003	22,716 (6.6)	22,423 (6.5)	0.068	40,494 (5.8)	35,952 (7.5)	Ceftriaxone(%)
0.012	129,223 (37.6)	127,194 (37.1)	0.244	212,453 (30.4)	202,093 (42)	Opioids(%)
0.005	12,958 (3.8)	12,627 (3.7)	0.006	23,616 (3.4)	16,745 (3.5)	Fibrates(%)
0.005	143,419 (41.8)	142,651 (41.6)	0.187	258,238 (36.9)	221,748 (46.1)	Acetaminophen(%)
<0.001	25,247 (7.4)	25,283 (7.4)	0.161	37,333 (5.3)	45,950 (9.5)	Codeine(%)
0.006	6,498 (1.9)	6,212 (1.8)	0.006	12,818 (1.8)	8,453 (1.8)	Amiodarone (%)
0.008	111 (<0.1)	162 (0.1)	0.027	134 (<0.1)	377 (0.1)	Anabolic Steroids(%)
0.008	5,884 (1.7)	5,551 (1.6)	0.035	13,194 (1.9)	6,951 (1.4)	Enalapril(%)
0.016	13,044 (3.8)	14,117 (4.1)	0.275	15,902 (2.3)	40,469 (8.4)	Systemic hormonal contraceptives(%)
0.013	10,733 (3.1)	11,547 (3.4)	0.264	12,041 (1.7)	34,123 (7.1)	Estrogens(%)
0.01	47,058 (13.7)	45,856 (13.4)	0.066	83,960 (12)	68,464 (14.2)	Furosemide(%)
0.005	51,508 (15)	50,947 (14.8)	0.218	73,235 (10.5)	86,883 (18.1)	Losartan(%)
0.006	20,925 (6.1)	21,449 (6.3)	0.205	29,189 (4.2)	44,597 (9.3)	Metronidazole(%)
0.004	5,419 (1.6)	5,568 (1.6)	0.046	10,000 (1.4)	9,734 (2)	Pyridoxine(%)
0.002	13,134 (3.8)	13,235 (3.9)	0.083	21,595 (3.1)	22,571 (4.7)	Ranitidine (%)
<0.001	35,567 (10.4)	35,546 (10.4)	0.206	48,753 (7)	63,194 (13.1)	Rosuvastatin(%)
0.105	42,135 (12.3)	31,028 (9)	0.123	86,389 (12.3)	41,369 (8.6)	Dipeptidyl Peptidase-4 inhibitors(%)
0.014	29,736 (8.7)	28,429 (8.3)	0.056	62,657 (9)	35,662 (7.4)	Smivastatin(%)
0.005	32,855 (9.6)	33,357 (9.7)	0.281	42,471 (6.1)	69,889 (14.5)	Tetracyclines(%)
0.001	32,707 (9.5)	32,845 (9.6)	0.218	45,106 (6.4)	61,863 (12.9)	Trimethoprim(%)
0.003	30,201 (8.8)	30,472 (8.9)	0.196	43,064 (6.2)	56,463 (11.7)	Sulfamethoxazole(%)

Supplementary Table S3: Diagnostic, Procedure, and Medication Codes Used for Query, Propensity Matching, or Outcomes

Associated Code	Search Terms
**ICD-10:** K85	Acute Pancreatitis*
**ICD-10:** K85.1	Biliary Acute Pancreatitis*
**ICD-10:** K86.1, K86.81	Chronic Pancreatitis*
**ICD-10 :** K86.3	Pancreatic Pseudocyst*
**ICD-10:** K86.2	Pancreatic Cyst*
**ICD-10:** C25	Pancreatic Adenocarcinoma*
**HCPS:** S8037, **SNOMED:** 450556004, 449395004, 38671800, 432110004, 427133003, 6157006, 71247002, 432876003, **CPT:** 1021432, 43237, 43277, 43265, 00732, 43276, 43275, 43274, 43262, 43262, 43264, 1007283, 1021432, 1021432, 1007934, 43263, 43278, 00732, **ICD-10 PCS:** 0F54, 0FT4, 0397T	MRCP, EUS, ERCP
**ICD-10:** E84, Q44, K85.8, Q45, K86.0, K83.01, K85.2, K86.9 **TNX Curated:** 9004**	Other causes of Pancreatitis Not-otherwise listed
**ICD-10:** Z98.84	Bariatric Surgery
**ICD-10:** F10	Alcohol Related Disorders
**LOINC:** 2469-5***	Autoimmune Pancreatitis
**ICD-10:** D37-D49, D00-D09, C73 - C96, C62-C69, C43-C58, C30-C41, C30-C39, C00-C26, C7B, C7A	Neoplasms
**ICD-10:** K80	Cholelithiasis
**ICD-10:** K87, K83.0, K81, K80.0, K8.01, K80.3, K80.4, K80.6, K82.0, K91.5, K91.86, K83.1, K83.2, K83.3, K83.4, K83.5, K83.8, K83.9<br>**CPT:** 1007869	Biliary Tract Diseases including Cholecystitis
**ICD-10:** E11, E11.31-E11.35, E11.4, E11.5	Type 2 Diabetes Mellitus with or without complications
**SNOMED:** 119916001, **CPT:** 1007914, 1007932, 1007934, 1007944, 48105 **ICD-10-PCS:** BF11, 0FBG, 0F54, 0FB4, 0FT4	Surgical Procedures on the Pancreas
**ICD-10:** I10	Hypertension
**ICD-10:** E78.4, E78.5	Hyperlipidemia
**ICD-10:** I70	Atherosclerosis
**ICD-10:** I73.9	Peripheral Vascular Disease
**ICD-10:** I63	Cerebral Infarction
**ICD-10:** I25.1	Coronary Artery Disease
**ICD-10:** F17	Tobacco Use Disorder
**ICD-10:** F12	Cannabis Use
**ICD-10:** N18	Chronic Kidney Disease
**ICD-10:** O00-O9A	Pregnant
**RxNorm:** 18330, 2020, 54552, 8700, 1102261, 10324, 2541, 52582, 6038, 6876, 7226, 10237	Medications with Cohort Frequency <0.1%****
**ICD-10:** Z79.890 **VA:** CN101, VT104, AM250 **ATC:** N02A, C10AB, A14A, G03A, G03C **RxNorm:** 161, 2670, 703, 4603, 52175, 6922, 9143, 301542, 36567, 10829, 10180, 7617, 2193	Medications with Frequency >0.1%****
**ATC:** A10BJ **RxNorm:** 1991302, 1551291, 60548, 1440051, 475968	Glucagon-like Protein-1 Receptor Agonists
**ATC:** A10BG **RxNorm:** 33738	Thiazolidinediones
**ATC:** A10BB	Sulfonylureas
**ATC:** A10BH	Dipeptidyl Peptidase 4 Inhibitors

**ATC:** A10BK **RxNorm:** 1373458, 1488564, 1545653, 1992672	Sodium-Glucose CoTransporter-2 Inhibitors

*After excluding all other causes of pancreatitis
** With Threshold of Triglycerides ever greater than 500 mg/dL
***With Threshold of IgG Subclass 4 ever > 280 mg/dL
****Medications with class 1a association with acute pancreatitis as Simons-Linares et al. 2019
ICD-10 = International Classification of Diseases Version 10, **PCS** = Procedure Coding System, **CPT** = Current Procedural Terminology, **ATC** = Anatomical Therapeutic Chemical Classification System, **LOINC** = Logical Observation Identifiers Names and Codes, **RxNorm** = US Normalized Drug Names **VA =** Veteran's Affairs Administration Drug Codes. **MRCP** = Magnetic Resonance Cholangiopancreatography, **EUS =** Endoscopic Ultrasound, **ERCP =** Endoscopic Retrograde Cholangiopancreatography.

Supplementary Table S4: Time-specific analyses of pancreatic cancer following GLP-1 RA versus SGLT2i initiation.

Hazard Ratio (95% CI)	Duration of follow-up after index-event	Outcome	Medication Type
0.954 (0.771 – 1.181)	1-year	Pancreatic Cancer	
0.981 (0.843 – 1.141)	3-years	Pancreatic Cancer	SGLT2i
1.057 (0.928 – 1.204)	10-years	Pancreatic Cancer	

Supplementary Table S5. STROBE Statement—Checklist for Cohort Studies

Location in Manuscript	STROBE Recommendation	Item No.
Title; Abstract	Identify study design in title/abstract; provide balanced summary	1
Introduction	Explain scientific background and rationale	2
Introduction: final paragraph	State specific objectives and hypotheses	3
Methods: Data Source and Study Design	Present key elements of study design early	4
Methods: Data Source and Study Design; Patient Selection and Cohort Construction	Describe setting, locations, and relevant dates, including period of exposure and follow up	5
Methods: Patient Selection and Cohort Construction; Exclusion Criteria	Eligibility criteria, participant selection	6a
Methods: Covariates and Propensity Score Matching; Figure 1; Supplemental Tables S1 and S2	Matching criteria and numbers matched	6b

Methods: Covariates; Study Outcomes; Supplemental Table S3	Clearly define exposures, outcomes, predictors, confounders, effect modifiers	7
Methods: Data Sources and Study Design; Covariates; Study Outcomes; Supplemental Table S3	Data sources and assessment methods	8
Methods: Exclusion Criteria; Propensity Score Matching; Sensitivity Analysis; Disucssion: Limitations	Describe efforts to address potential bias	9
Methods: Patient Selection and Cohort Construction; all eligible patients meeting prespecified criteria were included	Explain study size / rationale	10
Methods: Statistical Analysis	Handling of quantitative variables	11
Methods: Statistical Analysis; Propensity Score Matching	Describe all statistical methods including confounding control	12a
Methods: Sensitivity Analyses	Methods for subgroup analyses / interactions	12b
Methods: Statistical Analysis/ Missing Data	Explaining handling of missing data	12c
Methods: Study Outcomes and Statistical Analysis; Participants were censored at outcome occurrence; death, loss to follow-up, or end of data availability	Handling of loss to follow-up	12d
Methods: Sensitivity Analyses; Results: Sensitivity Analysis; Supplemental Table S4	Sensitivity analyses	12e
Results: Cohort Construction; Figure 1	Report numbers at each study stage	13a
Methods: Exclusion Criteria;	Give reasons for exclusion	13b
Figure 1	Flow diagram	13c
Results: Baseline Characteristics	Characteristics of study participants	14a
Methods & Results	Number with missing data	14b
Results/Supplementary Material	Summarize follow-up time	14c
Results: Primary and Sensitivity Analyses; Figure 2-3; Supplementary Material	Outcome events or summary measures	15
Results: Figure 2-3; hazard ratios with 95% confidence intervals	Provide unadjusted/adjusted estimates with precision	16a
Methods: Variable Definitions	Report category boundaries	16b

Results: Kaplan-Meier cumulative incidence estimates.	Translate estimates into absolute risk	16c
Results: Sensitivity Analyses; Figure3; Supplemental Table S4	Report other analyses performed, including subgroup and sensitivity analyses	17
Discussion: Summary of Findings	Summarize key results	18
Discussion: Limitations	Discuss limitations	19
Discussion: Interpretation and Conclusion	Interpret results cautiously	20
Discussion: Interpretation and Limitations	Discuss generalizability	21
Funding Statement	Funding sources and role	22
Institutional Review Board Statement	Ethical approval / IRB exemption	23

Supplemental Methods

1. **Propensity Score Matching:** In the TriNetX platform, this technique was implemented using one-to-one matching based on significant covariates through greedy nearest neighbor algorithms, and a caliper width fixed at 0.1 pooled standard deviations (SDs). Comparisons of characters that had a standardized mean difference of less than 0.1 was considered indicative of proper matching.
2. **Kaplan-Meier Analysis:** We performed Kaplan-Meier analysis on the propensity-matched cohorts within the TriNetX platform as outlined by Austin et al., 2014 (1). Patients were followed until an outcome occurred or were censored after the last available data or death.
3. **Log-Rank Testing:** Log-rank tests were employed on the TriNetX platform to evaluate the statistical differences in time to an event for every cohort. A rejection of the log-rank test null hypothesis signifies significant differences in survival curves.
4. **Benjamini & Hochberg Correction Method:** We use the Benjamini & Hochberg technique to control for the False Discovery Rate(2). T his approach ranks p-values and modifies them to recognize significant findings, thereby maintaining control over incorrect discoveries. We favored this technique over the Bonferroni correction, which presumes independence between outcomes, given that acute pancreatitis likely impacts the risk of other assessed outcomes.
5. **Univariate Cox-Proportional Hazards Ratios Model:** Hazard ratios (HRs) were calculated in the TriNetX platform.
6. **Schoenfeld Residual:** We verified the Cox proportional hazards model via scaled Schoenfeld residuals calculated in the TriNetX platform. The proportional hazards assumption assumes that the covariates' effect remains unchanged over time. A violation of this assumption occurs when the null hypothesis is rejected, suggesting significant changes over time.
7. Time-horizon sensitivity analysis **Analyses:** For outcomes that violated the proportional hazards assumption, we carried out time-horizon sensitivity analysis analyses at predetermined time points such as 1,3 and 10- years after the index event. The time periods were selected based on the timeline of the expected outcomes and examines if the hazard ratios remained similar over time.
